# Home-Based Nonimmersive Virtual Reality Training After Discharge From Inpatient or Outpatient Stroke Rehabilitation: Parallel Feasibility Randomized Controlled Trial

**DOI:** 10.2196/64729

**Published:** 2025-03-28

**Authors:** Lisa Sheehy, Anne Taillon-Hobson, Heidi Sveistrup, Martin Bilodeau, Christine Yang, Vivian Welch, Hillel Finestone

**Affiliations:** 1 Bruyère Health Research Institute Ottawa, ON Canada; 2 Faculty of Health Sciences Schools of Rehabilitation Sciences and Human Kinetics University of Ottawa Ottawa, ON Canada; 3 Department of Systems and Computer Engineering Carleton University Ottawa, ON Canada; 4 Bruyère Health Ottawa, ON Canada; 5 Division of Physical Medicine and Rehabilitation Faculty of Medicine University of Ottawa Ottawa, ON Canada; 6 Faculty of Medicine School of Epidemiology and Public Health University of Ottawa Ottawa, ON Canada

**Keywords:** virtual reality, telerehabilitation, stroke, home, exercises, physical activity, physiotherapy, exergames, rehabilitation intensity, randomized controlled feasibility trial, motor, movement, patient care, patient engagement, health intervention, stroke rehabilitation, interactive games, game therapy, interactive therapy, rehabilitation

## Abstract

**Background:**

Nonimmersive virtual reality training (NIVRT) can be used to continue rehabilitative exercise for stroke recovery at home after discharge from inpatient or outpatient therapy.

**Objective:**

The objectives of this randomized controlled feasibility trial were to assess home-based NIVRT as telerehabilitation with patients living with stroke, and its potential to improve standing function and gait.

**Methods:**

Patients approaching discharge from inpatient or outpatient stroke rehabilitation were randomly allocated to NIVRT or iPad interventions. NIVRT provided interactive games and exercises designed to improve balance, stepping, and aerobic capacity. iPad apps addressed cognition and fine motor skills. Participants were visited in their homes by a physiotherapist, taught to use the program, and asked to do 30 minutes of exercise 5 days a week for 6 weeks, asynchronously. Feasibility was assessed by measuring recruitment, adherence, ability to set up and learn NIVRT, enjoyment, intent to continue, perception of impact, and safety. Participants completed assessments of standing balance, gait, and general function, before and after the intervention, by a blinded assessor.

**Results:**

NIVRT participants (n=11; 10 male participants; mean age 64, SD 12 years) did an average of 26 sessions (total 700 minutes), while iPad participants (n=9; 6 male participants; mean age 61, SD 20 years) did an average of 33 sessions (total 1241 minutes). Space was tight in 5 homes. All but 1 participant learned NIVRT and progressed. Most enjoyed it and felt that it improved their recovery. There were no serious adverse events. Most assessments showed improvement over time for both groups.

**Conclusions:**

Home-based NIVRT is safe and feasible to continue rehabilitative exercise after discharge. More research on efficacy and effectiveness in this population is required.

**Trial Registration:**

ClinicalTrials.gov NCT03261713; https://clinicaltrials.gov/study/NCT03261713

**International Registered Report Identifier (IRRID):**

RR2-10.1186/s13063-019-3438-9

## Introduction

More than 108,000 individuals experience a stroke each year in Canada [1], with roughly 40% having moderate to severe disability requiring intense therapeutic intervention [2]. Rehabilitation is essential to maximize recovery after stroke [3]. Substantial evidence suggests that greater benefit is obtained by providing a greater intensity of rehabilitation [4]. Unfortunately, many patients complete their formal rehabilitation before recovery is complete [5]. When a patient is discharged home, maintaining a regimen of therapeutic exercise until recovery has plateaued is essential. However, outpatient and community-based rehabilitation may be inaccessible for reasons such as lack of programs, transportation difficulties, inclement weather, fatigue, or financial limitations [6]. For these patients, home-based therapy may be preferred. Home-based care (eg, doing a prescribed number of isolated exercises every day, intended to improve muscle strength, coordination, balance, and gait [7]) does not lead to worse outcomes compared to institution-based care [8].

Despite its importance, home-based exercise can be dull, and patients tend to reduce their adherence over time [7]. One rehabilitation approach that has been shown to mitigate these obstacles is nonimmersive virtual reality training (NIVRT, also called exergaming), in which a camera, coupled with computer software, allows a patient to interact with a game or exercise presented on a TV. NIVRT is convenient, engaging, and motivating [9], and may encourage the user to increase their rehabilitation intensity, either by doing more repetitions per session or by doing more sessions per week [10,11]. NIVIRT can be used as a form of asynchronous telerehabilitation, in which the therapy is done without the therapist being present, either in-person or virtually [6].

Telerehabilitation using NIVRT has been shown to be as good as conventional therapy for the rehabilitation of upper extremity deficits, activities of daily living, and postural balance after stroke [12-14]. One meta-analysis found that NIVRT-based telerehabilitation improved upper extremity function more than an active control, as assessed by the Fugl-Meyer Assessment [15]. Safety, feasibility, and enjoyment have been shown for upper extremity home-based NIVRT [13,16], and there is budding evidence of feasibility for lower extremity motor recovery, gait, and balance in subacute and chronic stroke [16-18]. Lee et al [17] reported a small but significant benefit to doing balance and gait-related NIVRT via telerehabilitation, with moderate usability (the exercises were easy to learn but technical issues were common) and no reported falls. Users in several studies reported NIVRT to be motivating [16]. In addition, the use of NIVRT (not telerehabilitation) for the improvement of gait in chronic stroke is enjoyable and safe [19]. Laver et al [20] reported insufficient evidence to determine the effects of telerehabilitation on improvement in mobility after stroke, although Hao et al [15], in their updated meta-analysis, found that there was no difference between NIVRT and traditional therapy in gait speed, walking endurance, balance, and functional mobility. However, Hao et al [15] only included 5 studies in their analysis. Of the 5 studies, one was conducted in a simulated home environment at the hospital, one compared telerehabilitation using NIVRT to no home-based therapy, one compared the NIVRT telerehabilitation intervention with a similar clinic-based one or conventional hospital-based therapy, one compared the same NIVRT performed in the clinic and the home, and one recruited people living with chronic stroke living in long-term care, and used an active control group [21-25]. Compared to upper extremity therapy, there are few studies investigating the use of NIVRT as telerehabilitation for balance, gait, and function, particularly in the subacute phase after stroke, when rehabilitation is more likely to provide benefits [26].

The purpose of this study was to investigate if an NIVRT-based telerehabilitation program for balance, gait, and function, provided shortly after discharge from inpatient or outpatient rehabilitation, is acceptable and has the potential for benefit. Before creating a strong protocol for a definitive efficacy study, feasibility, safety, and acceptance need to be clarified [27]. The potential efficacy of NIVRT can be tested with a small sample, which will also assess the selected outcome measures and provide data for future sample size estimation. The results of this randomized controlled feasibility trial will inform the methods and sample size of a future randomized controlled trial.

The primary objective was to assess the feasibility of using NIVRT as telerehabilitation with patients poststroke. There were five components to this feasibility objective:

- To estimate the recruitment of participants into the study
- To assess the ability of participants and their study partners to install and use the NIVRT technology within their homes
- To assess the ability of participants to learn NIVRT given the training provided and to adhere to the exercise protocol (retention
- To determine the safety of NIVRT
- To determine the acceptability of NIVRT for stroke recovery (enjoyment, perceived efficacy)

The second objective was to assess the potential of telerehabilitation using NIVRT with patients living with stroke to maintain or improve physical outcomes of standing balance, gait, general function, and community integration after discharge from inpatient or outpatient rehabilitation, compared to those who performed a program of iPad apps designed for fine hand motor skills and cognitive training.

## Methods

### Study Design

This study was a prospective, single-site, assessor-blinded, parallel-group randomized controlled feasibility trial. Participants were randomized to NIVRT or iPad (active control intervention). The protocol for this study has been published [28]. The iPad control group was chosen to ensure that all participants had equal contact time with the research team and approximately equal time spent doing an engaging activity, but the control activity would not be expected to have any direct influence on the physical outcomes of balance, gait, and function. Using an active control group also aided in recruitment [28]. CONSORT (Consolidated Standards of Reporting Trials) guidelines for randomized pilot and feasibility trials were followed [29,30]. The CONSORT checklist is available in Multimedia Appendix 1.

### Participants

In total, 20 participants were recruited from a tertiary-care urban hospital located in Ontario, Canada, between July 2017 and October 2018. Participants were recommended by staff physiotherapists. Because this was a feasibility study rather than an efficacy study, no sample size calculation was performed. Instead, the sample size was determined based on recruitment expectations, available funding, and the potential to address the feasibility outcomes.

Patients were eligible if they were approaching discharge from either inpatient or outpatient stroke rehabilitation, with the intent that they would participate post discharge. While there was no eligibility criterion regarding the chronicity of stroke, inpatient and outpatient stroke rehabilitation at this facility was generally reserved for patients in the subacute phase after stroke. Potential participants must (1) have had an ischemic or hemorrhagic stroke resulting in physical impairment, (2) be cognitively able to provide informed consent and therefore presumed able to learn NIVRT, (3) be able to stand independently for at least 2 minutes and able to perform mild to moderate exercise, (4) have a study partner (a family member or friend who would be trained to use NIVRT along with the participant and be present in the home during NIVRT), (5) be able to read, speak, and understand English, (6) have no plans to travel away from home for more than 2 days a week, and (7) have enough space in their home to do NIVRT. Patients were excluded if they had an unstable medical condition, seizures, or vertigo. Participants were recruited by a research assistant, who randomized them to NIVRT or iPad groups in a 1:1 ratio with permuted blocks, using a computer-based randomization platform (Sealed Envelope). Allocation was concealed from the assessment physiotherapist (who did the assessments) and from other participants. It was impossible to blind the participants as to their allocation.

Age, sex, BMI, hand dominance, weeks since stroke, type, and side of stroke were recorded. The Motivation for Physical Activity Question (which has high levels of reliability) [31] was administered during the preassessment and the Physical Activity Enjoyment Scale (PACES) (which has satisfactory or better validity and test-retest reliability when administered to older adults) [32,33] was administered at the postassessment to help describe participant samples.

### Interventions

**Figure 1:**
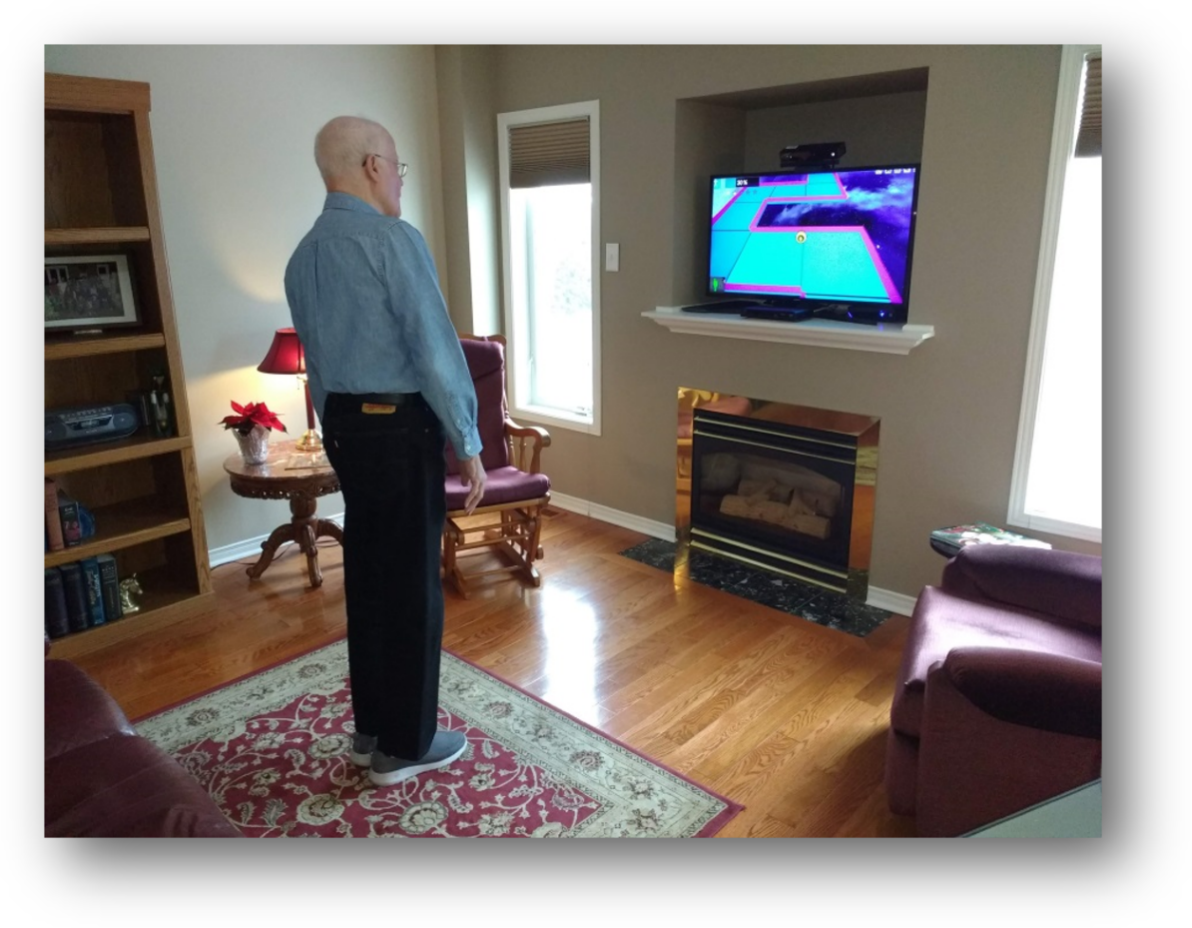
Participant performing nonimmersive virtual reality training in his home.

NIVRT was provided using the Jintronix NIVRT platform. Infrared sensors combined with Microsoft software enabled a Kinect camera (Microsoft Canada Co) to track the participant’s movements, allowing them to interact with a game or exercise presented on a TV screen (Figure 1). Games and exercises addressed rehabilitative goals of improving balance (eg, slalom skiing, moving a ball around a maze), range of motion (eg, arm circles), strength (eg, sit-to-stand), reaching (eg, “planting” and “harvesting” tomatoes), stepping (eg, whack-a-mole), and aerobic capacity (eg, marching on the spot). Parameters such as range of motion, distance, speed, or accuracy were selected based on the participant’s physical abilities, fall risk, tolerance, and rehabilitation goals. For example, for slalom skiing, the speed at which the gates appeared, the distance to lean, the proportion of right to left leans, the addition of banners to squat under, and the number of gates could all be customized. For more details, see Sheehy et al [28].

iPads (Apple) for participants in the iPad group were loaded with apps designed to address memory and cognition, visual tracking and scanning, and fine motor skills. There was no specific selection of apps for all iPad group participants. The iPad intervention provided the participants with an activity that was engaging and took the equivalent amount of time to the NIVRT program; however, the apps were not expected to impact balance, gait, and mobility.

### Outcome Measures

To address the first feasibility objective, the number of participants recruited over 15 months was recorded and presented relative to the number of patients admitted for stroke rehabilitation.

The ability of participants and their study partners to install and use the NIVRT technology and to learn the individual NIVRT games and exercises was assessed through comments provided by the participant and study partner in a logbook, field notes recorded weekly by the intervention physiotherapist, and during a semistructured interview (Multimedia Appendix 2), administered to the participant and study partner at the end of their participation. Questions regarding the acceptability of NIVRT were included in the interview and assessed with the PACES.

Adherence to the protocol and progression were monitored asynchronously by tracking the use of NIVRT (using data stored within the Jintronix platform) and the iPad (using a logbook). If a majority of participants performed less than 450 minutes of NIVRT (75 minutes per week) over the course of the trial, the use of NIVRT for motivation would be reconsidered. If no progression was observed, the potential factors responsible (eg, poor ability to learn) would be deliberated. Retention was tracked using a spreadsheet; a loss of greater than 25% would suggest that the protocol should be reviewed prior to launching a future definitive randomized controlled trial.

The intervention physiotherapist inquired about major adverse events (eg, death or injuries serious enough to require withdrawal from the study) and minor adverse events (eg, mild joint or muscle soreness that responded to changes in the intervention) at each contact with the participant. The occurrence of major adverse events would indicate that telerehabilitation provided via NIVRT according to the protocol is too risky to continue.

To address the second objective, the following tests were administered in-person by the assessment physiotherapist, all of which have adequate to excellent reliability, validity, and responsiveness to change (unless noted below):

1. Berg Balance Scale (BBS), which tests balance and mobility [34-36].
2. Timed Up and Go (TUG) test, which tests ambulatory function. A total of 3 variations were administered: original, manual dual-task (carrying a cup of water), and cognitive dual-task (counting backward in 7’s from 100) [37-40].
3. Five Times Sit-To-Stand (FTSTS) test, which assesses lower-extremity strength [41,42]. Responsiveness to change has not been confirmed in stroke populations.
4. Community Balance and Mobility (CB&M) Scale, which tests more difficult tasks relevant to community ambulation [43,44].
5. Stroke Impact Scale (SIS) (v3.0), which assesses 59 components of health status, each scored from 2-5 for a maximum total of 295 (higher scores equal less impact of the stroke), as well as a separate visual scale measuring recovery from 0 (no recovery) to 100 (full recovery) [45].
6. Community Integration Questionnaire (CIQ), which assesses integration in home, social, and productivity domains with a maximum total of 29 (higher score equals greater integration) [46,47].

These final two outcome measures were included for their potential to assess the impact of improved balance, gait, and mobility on quality of life and community integration.

### Procedures

After consenting, participants first attended two one-hour preassessment sessions at the hospital, administered by the assessment physiotherapist who was blinded to intervention allocation. Upon completion of the preassessments, each participant was randomized to either the NIVRT or iPad group by the intervention physiotherapist, who was blinded to the preassessment results. The intervention physiotherapist created a customized program of NIVRT or iPad apps, in consultation with the participant’s clinical physiotherapist and occupational therapist, and within 5 days visited the home of the participant and their study partner. For NIVRT group participants, the NIVRT equipment was installed in the home and the participant and study partner were trained on its use (including safety considerations and what to do should a fall or injury occur). The study partner was instructed to assist with technical requirements, motivation, and supervision as needed, but not to physically assist or guard the participant during exercises. For iPad group participants, the iPad was brought to the home and the participant and study partner were trained on the apps. For both groups, written material supplemented the training, and a logbook was provided to write down comments and the time spent doing each app (iPad users only). Participants were requested to perform the intervention 30 minutes a day, 5 days a week, for 6 weeks, asynchronously, without direct supervision from the intervention physiotherapist.

The intervention physiotherapist contacted each participant by phone at least weekly to review the programs, answer questions, and suggest progressions. The NIVRT programs were modified and progressed remotely if participants consistently achieved 100% on an activity, found the activity too easy, became bored, or wished to work on different therapeutic goals. Suggestions for new or modified iPad apps were made if participants became bored, found the apps too easy, or wished to focus on different tasks. Field notes were recorded after each interaction.

After 6 weeks of intervention, participants returned to the hospital for the postassessment, which was completed in one session. Participants in the iPad group gave the iPad to the intervention PT before their postassessment appointment. For NIVRT participants, the intervention PT visited the home to remove the equipment.

### Data Analysis

Participant demographics (including the Motivation for Physical Activity Question and PACES) were described using means and SDs, or proportions, and compared using independent samples *t* tests, Fisher exact tests, and Mann-Whitney U tests. Quantitative outcome measures were described using means and 95% CIs and SDs. Missing data were addressed by removing cases analysis by analysis. Scales were compared using mixed-model analyses of variance (within-group factor: time; between-group factor: group; interaction: time × group). If the interaction was insignificant, the interaction was removed, and the main effects of each factor were assessed. Assumptions (eg, presence of outliers, normal distribution of data, homogeneity of variances, and homogeneity of covariances) were assessed before the analyses of variance were carried out [48]. Data analyses were performed with SPSS (version 28.0.1.1; IBM Corp).

Qualitative data were analyzed using thematic analysis following the model provided by Castleberry and Nolen [49]. Interviews were transcribed. They were compiled, along with logbook entries and intervention physiotherapist records of contact with participants, then disassembled (ie, coded) by 2 research team members and reassembled into themes, using an iterative process begun with deductive reasoning (using the objectives as premises) and including inductive reasoning as required [49,50]. The 2 research team members did this independently and then conferred to review and interpret the themes. Each team member engaged in a reflexivity exercise before beginning the analysis. Disagreements were moderated by a third team member until consensus was reached.

### Ethical Considerations

Research ethics approval was obtained from the Bruyère Health Research Institute (M16-17-013) and the University of Ottawa (A01-15-03) research ethics boards. The trial was registered at ClinicalTrials.gov (NCT03261713). Participants and their study partners provided written informed consent. Data were deidentified and associated with participant study IDs only. Participants did not receive any compensation other than travel expenses (eg. parking fees or return taxi fare or bus tickets; plus a CAD $5 (US $3.50) coffee shop voucher if the visit was over the lunch hour) for the postassessment sessions.

## Results

### Participants

**Figure 2:**
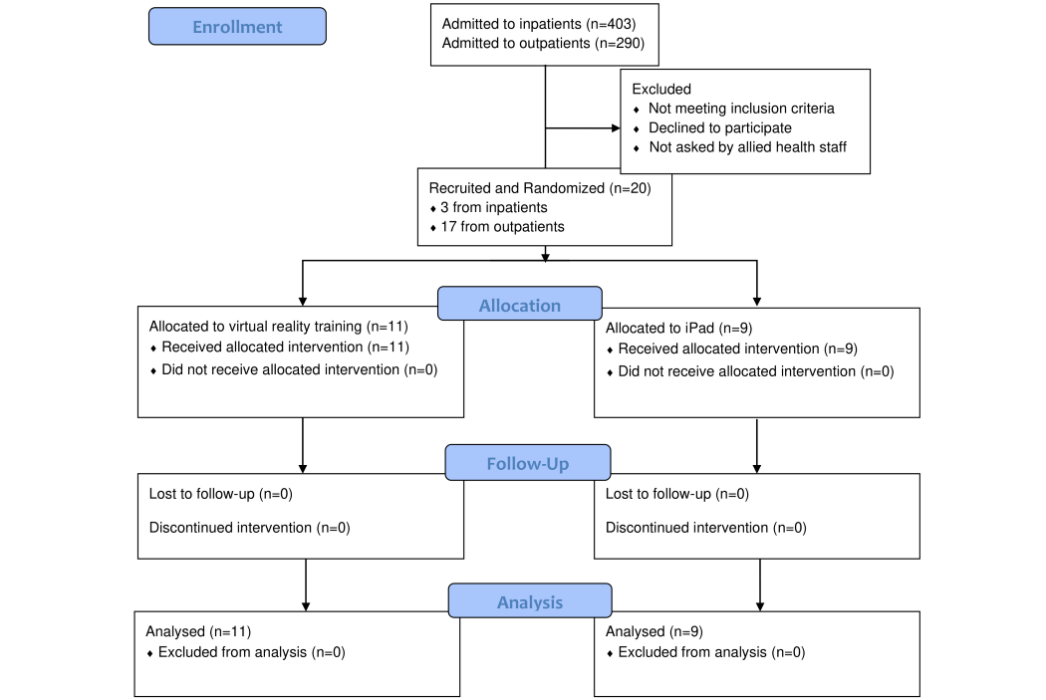
CONSORT (Consolidated Standards of Reporting Trials) 2010 flow diagram.

Of the 20 participants recruited, 17 participants were recruited through the outpatient service and 3 from the inpatient service; all completed the study. From July 1, 2017, to September 30, 2018, there were 403 inpatient and 290 outpatient admissions. See Figure 2 for the CONSORT flow diagram, Table 1 for a summary of participant demographics, and Multimedia Appendix 3 for details of the participants. There was one outlier, in which a participant in the iPad group was enrolled 81 weeks post stroke. Otherwise, there were no differences between groups.

**Table 1:** Summary of participant demographics.

Demographics		Nonimmersive virtual reality training group (n=11)	iPad group (n=9)
Age (years), mean (SD)		63.7 (11.9)	61.0 (20.3)
**Sex, n**			
	Male	10	6
	Female	1	3
BMI (kg/m^2^), mean (SD)		24.9 (3.3)	27.5^a^ (3.9)
**Hand dominance, n**			
	Right	9	8
	Left	2	1
Weeks since stroke, mean (SD)		15.1 (7.9)	16.9^a^ (8.1)^b^
**Type of stroke, n**			
	Ischemic	8	9
	Hemorrhagic	3	0
**Side of stroke, n**			
	Right	6	4
	Left	5	5
Motivation for physical activity question, mean (range)		5.4 (0-6)	5.8 (4-6)
Physical activity enjoyment scale, mean (SD)		92.5 (19.9)	101.0 (19.6)

^a^Based on 8 participants.

^b^There was one outlier who enrolled 81 weeks after their stroke. If their data are included, the average weeks since stroke was 24.0 (SD 22.7).

The qualitative data were used to contextualize the quantitative data associated with each component of the primary (feasibility) objective. The themes agreed upon by the research team were installation and technology (related to primary objective 2), ability to learn and progress (primary objective 3), games and glitches (primary objective 3), perceived usefulness (primary objective 5), enjoyment (primary objective 5), and interest to use again (primary objective 5).

### Primary (Feasibility) Objective 1: Estimation of Recruitment of Participants

Approximately 1% of inpatients (3/403) and 6% of outpatients (17/290) were recruited. Inpatients were hesitant to participate as they felt anxious and overwhelmed around the time of discharge. Physiotherapists and occupational therapists in the outpatient stroke program used NIVRT with some of their patients; however, no participant mentioned that they had used NIVRT before. One participant mentioned that it would have been helpful to begin using NIVRT as an inpatient to develop the routine of using it.

### Primary (Feasibility) Objective 2: Ability to Install and Use Nonimmersive Virtual Reality Training Technology

The NIVRT system was successfully installed in all participant’s homes; however, some issues were identified. One issue was space. Of the 11 participants using NIVRT, 4 had it installed in their living room, 3 in their basement, 2 in their den, and 2 in their bedroom. The choice was informed by the available space and the proximity of the participant’s TV. One participant wanted to keep the computer equipment out of the reach of grandchildren. All participants used their own TV, which was mounted on a wall (5 participants) or a horizontal surface (6 participants). A total of 5 participants had less than 2.0 m of unobstructed space in front of the camera, which led to difficulties with the camera properly tracking the movement of the lower extremities. One TV did not have an accessible HDMI port; an HDMI-to-AV converter was used, resulting in mediocre picture quality. All participants had Wi-Fi in their homes.

Most participants managed the use of the computer well, although one participant had difficulties with turning the computer on and off and using a mouse. One participant mentioned difficulty with typing and suggested a one-piece system with one-button accessibility. The computers were set up to not require passwords; however, unexpected issues arose, such as power outages, computer sound not working, and a Windows feature update that caused incompatibility with the Jintronix software for a time. All issues but one (the need for the HDMI-to-AV converter) were resolved remotely.

The requirement for the participant and study partner to speak English was important, as several participants and study partners had difficulty communicating the intricacies of computer set-up and NIVRT (or iPad) use.

### Primary (Feasibility) Objective 3: Ability to Learn Nonimmersive Virtual Reality Training and Adhere to the Exercise Protocol

All participants but one learned to use NIVRT and progressed throughout the study. The single participant had poor motivation, despite encouragement from their study partner, and only completed 5 NIVRT sessions. Participants provided a variety of comments on their perceptions of the difficulty of the program. Some felt that it was too easy at times while others found that it was initially difficult but got easier over time.

**Figure 3:**
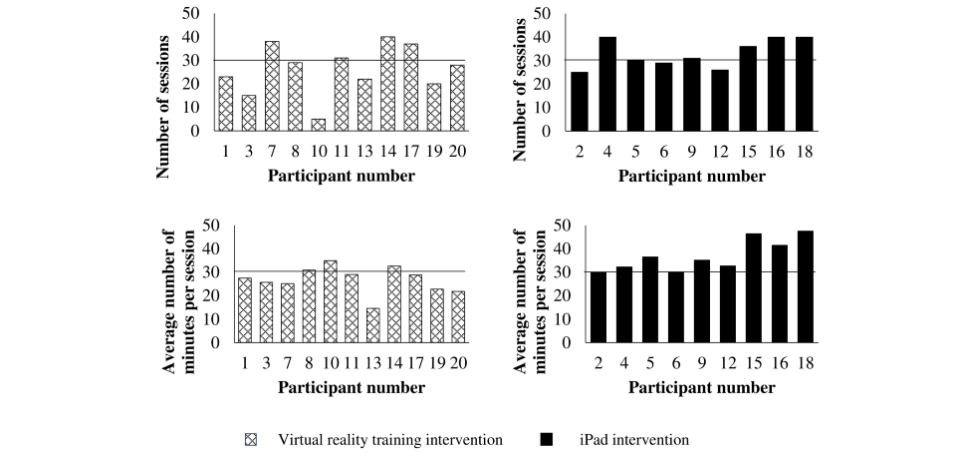
Number of sessions and average number of minutes of exercise per session for each participant. Horizontal black lines indicate the requested number of sessions or minutes per session.

All participants completed the study. Overall adherence to the program (30 minutes of intervention 5 days per week for 6 weeks for a total of 30 sessions and 900 minutes of therapy) was excellent. NIVRT participants did an average of 26 sessions with a total of 700 minutes while iPad participants did an average of 33 sessions with a total of 1241 minutes (Figure 3).

Several comments within the “games and glitch” theme related to the participants’ interaction with the NIVRT. Most participants commented that the Kinect camera had difficulty tracking their movements. While participants enjoyed games with direct feedback (eg, kicking a soccer ball), some games were described as “frustrating” (eg, putting cutlery in a drawer, which required advanced visuospatial skills). Many participants found the scoring confusing (eg, scoring did not reflect improvement) and the feedback annoying (eg, even when the exercise was done incorrectly, the system said “nice job”). One participant mentioned that they did not like the playback feature (after an exercise, the system replayed a short video of their performance).

### Primary (Feasibility) Objective 4: Safety of Nonimmersive Virtual Reality Training

There were no serious adverse events. There were 3 minor adverse events. One participant complained of shoulder pain for 20-30 minutes after doing NIVRT, due to “frozen shoulder.” They continued the program, adjusting as necessary. One participant suffered from a flare-up of gout, which was successfully treated, after which they were able to return to the program. One participant reported fatigue if they did NIVRT on the same day as other therapies.

### Primary (Feasibility) Objective 5: Acceptability of Nonimmersive Virtual Reality Training

Most participants enjoyed doing the NIVRT. Two participants said that while it was initially interesting and fun, later it became more like a chore. Others described NIVRT as “boring,” “simple,” and “convenient.” A total of 3 participants would have liked an in-person or group component to the program.

Participants and study partners commented in detail on the specific games and exercises. There was a wide variety of opinions regarding which games were enjoyable. Many liked exercises that focused on strengthening, balance, coordination, weight shift, range of motion, and stretching, while others specifically did not like the strengthening games.

Almost all participants reported that NIVRT was useful and improved their recovery. Notably, 1 participant reported improved gait, independence, and strength, and 2 mentioned improvements in daily tasks like putting on their pants and doing bilateral upper extremity activities. Some mentioned that NIVRT was motivating and that scheduling the NIVRT encouraged self-discipline to continue exercising after discharge. On the other hand, 2 participants found that NIVRT was more difficult to do than physiotherapy since there was no accountability. Several participants commented that NIVRT was a useful tool to improve recovery, but should be combined with walking, physiotherapy, and specific hand exercises (NIVRT does not focus on the hand). A total of 2 participants found that 5 times a week was excessive. When asked, 6 participants (out of 11) or their study partners were interested in continuing NIVRT. A single participant shared that they would prefer activities that garner a sense of community.

### Second Objective: Potential of Nonimmersive Virtual Reality Training to Improve Physical Outcomes and Community Integration

**Figure 4:**
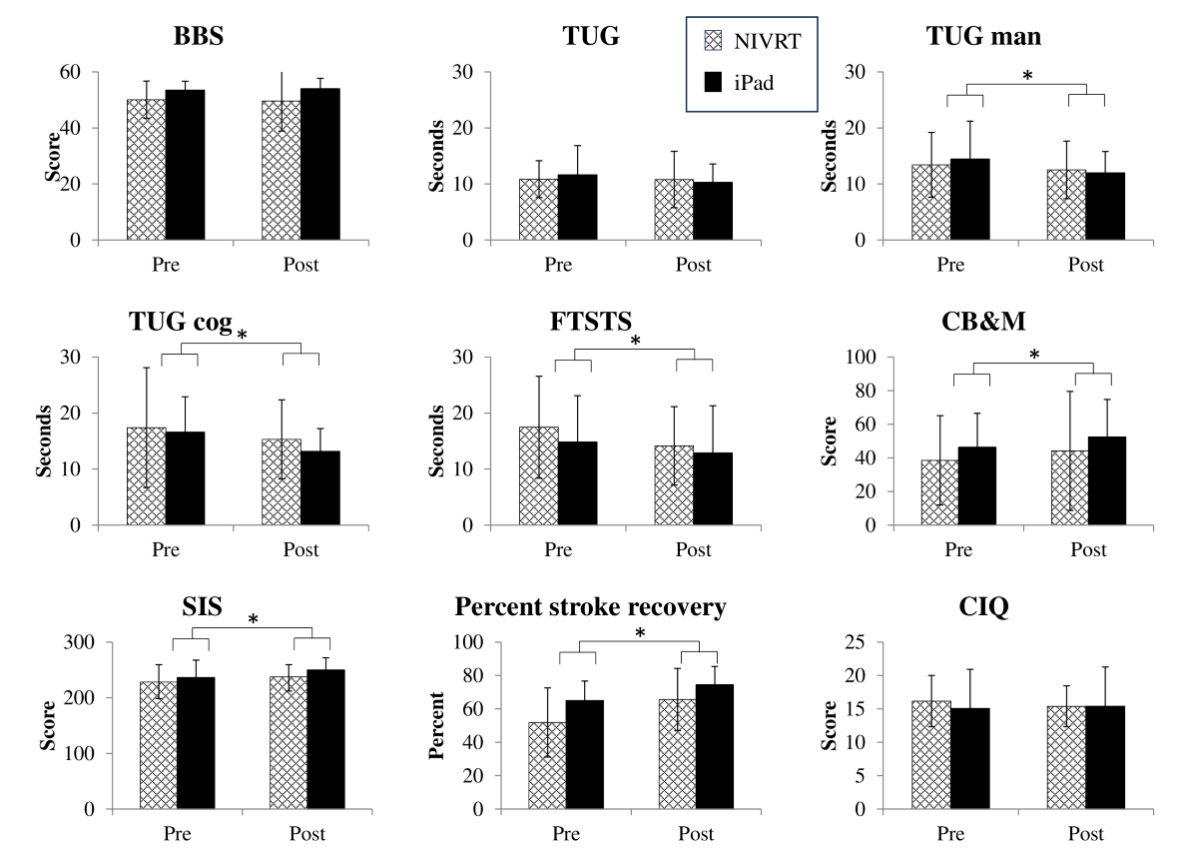
Physical outcome measures over the 6-week intervention period, presented as means and SDs. Comparisons with * are statistically significant for the main effect of change over time. BBS: Berg Balance Scale; CB&M: Community Balance and Mobility Scale; CIQ: Community Integration Questionnaire; FTSTS: Five Times Sit-To-Stand test; NIVRT: nonimmersive virtual reality training; SIS: Stroke Impact Scale; TUG: Timed Up and Go test; TUG cog: Timed Up and Go cognitive dual-task test; TUG man: Timed Up and Go manual dual-task test.

The results regarding the potential for NIVRT to affect physical outcomes and community integration are found in Table 2 and Figure 4. All assumptions were met except that the Levene test of homogeneity of variance for the post assessments of the CB&M scale was significant (*P*=.03), and there was one legitimate outlier on the BBS, which slightly affected the normal distribution and the homogeneity of variance for the preassessment. Since the deviations were small, parametric testing was performed as planned. There were no statistically significant interactions between group and time (ie, the intervention did not affect one group more than the other), and no statistically significant differences between groups. However, when the groups were combined (main effect of time), there was an improvement over the 6-week intervention period for several outcome measures.

**Table 2:** Outcome measures over the 6-week intervention period, presented as means and 95% CIs, with mixed-model analysis of variance results for interaction, group, and time.

Outcome measure	Nonimmersive virtual reality training, mean (95% CI)				iPad, mean (95% CI)				Interaction *F* test (*df*)		*P* value		Group main effect *F* test (*df*)		*P* value		Time main effect *F* test (*df*)		*P* value
	Pre	Post	Participants, n	Pre		Post	Participants, n												
BBS^a^	50.1 (46.7-53.5)	49.6 (44.3-54.9)	11	53.4 (49.7-57.2)		54.0 (48.1-59.9)	9	0.273 (1)		.61		1.637 (1)		.22		0.003 (1)		.96	
TUG^b^ original	10.9 (7.8-14.0)	10.8 (7.8-13.8)	9	11.6 (8.5-14.7)		10.3 (7.3-13.3)	9	0.845 (1)		.37		0.003 (1)		.95		1.028 (1)		.33	
TUG man^c^	13.4 (9.0 -17.9)	12.5 (9.3-15.7)	9	14.4 (10.0 -18.9)		12.0 (8.8-15.2)	9	1.738 (1)		.21		0.009 (1)		.92		8.194 (1)		.01 ^d^	
TUG cog^e^	17.4 (11.2-23.6)	15.3 (11.2-19.4)	9	16.6 (10.4-22.8)		13.2 (9.1-17.2)	9	0.625 (1)		.44		0.186 (1)		.67		11.043 (1)		.004^d^	
FTSTS^f^	17.5 (11.7-23.3)	14.2 (9.0 -19.3)	10	14.8 (8.7-21.0)		12.9 (7.4-18.3)	9	0.626 (1)		.44		0.286 (1)		.60		9.866 (1)		.006^d^	
CB&M^g^	38.5 (23.4-53.7)	44.2 (25.0-63.4)	11	46.3 (29.5-63.1)		52.4 (31.2-73.6)	9	0.007 (1)		.93		0.451 (1)		.51		4.440 (1)		.05^d^	
SIS^h^	228.1 (206.5-249.7)	237.6 (220.8-254.3)	9	236.1 (214.5-257.7)		249.9 (233.1-266.6)	9	0.355 (1)		.56		0.676 (1)		.42		10.202 (1)		.006^d^	
% Recovery (part of the SIS)	51.9 (39.4-64.3)	65.6 (54.3-77.0)	8	65.0 (53.3-76.7)		74.4 (63.8-85.1)	9	0.732 (1)		.41		2.288 (1)		.15		21.232 (1)		<.001^d^	
CIQ^i^	16.2 (12.7-19.6)	15.4 (12.1-18.7)	9	15.1 (11.4-18.8)		15.4 (11.9-18.8)	8	2.902 (1)		.11		0.060 (1)		.81		0.528 (1)		.48	

^a^BBS: Berg Balance Scale.

^b^TUG: Timed Up and Go test.

^c^TUG man: Timed Up and Go manual dual-task test.

^d^Statistical significance.

^e^TUG cog: Timed Up and Go cognitive dual-task test.

^f^FTSTS: Five Times Sit-To-Stand test.

^g^CB&M: Community Balance and Mobility.

^h^SIS: Stroke Impact Scale.

^i^CIQ: Community Integration Questionnaire.

## Discussion

### Principal Findings

This study shows that NIVRT provided via telerehabilitation is a feasible way for individuals who have recently had a stroke to continue with rehabilitative exercise focusing on lower-extremity function, balance, and gait at home. The results of this study will inform the methods and sample size of a future randomized controlled trial on the use of NIVRT in community-based stroke recovery.

A majority of the participant sample identified as male. Females and women are frequently underrepresented in stroke rehabilitation studies, which may limit the generalizability of the outcomes [51,52]. Female survivors of stroke show less functional recovery than males and experience greater barriers to regaining participation poststroke [53]. It is possible that existing negative attitudes and beliefs of women patients with stroke towards NIVRT affected their interest in the study [51]. Wiley et al [54] emphasized the importance of reinforcing positive expectations toward exercise after stroke for all genders. It is important to review eligibility criteria to avoid gender bias and provide recruitment strategies and resources for future NIVRT studies that are appealing to women and encourage their participation [51]. Maintaining a screening log that includes gender would also alert research team members if recruitment strategies need to be adjusted [51].

Only 1%-6% of patients were eligible and interested in the research program, highlighting several items to be considered while planning a definitive study and implementing NIVRT into practice. If patients had the opportunity to use NIVRT in the hospital setting, this might increase their comfort using the technology in the home [55]. The emphasis on remote care and telemedicine during and since the COVID-19 pandemic shutdowns may help future recruitment. Tracking every admission rather than relying on clinicians to suggest potential participants might lead to greater recruitment as well since gatekeeping by clinicians has been found to be a barrier to recruitment [56]. Including patient partners to help set priorities, inform the methods of the study, and review patient-facing materials for simplicity and ease of understanding may help recruitment in future studies and is suggested to improve the relevance of health research [57].

Inpatient and outpatient stroke rehabilitation is generally targeted towards subacute stroke; however, we did have one participant in the chronic stage. In a future definitive study, the stage of stroke recovery should be included as an eligibility criterion.

While all participants were able to have NIVRT installed in their homes and use the technology, certain characteristics of the participants and their homes were necessary for the success of using NIVRT. An unobstructed area of 2.0 m by 1.5 m was essential for NIVRT. Obstructions within that space prevented the Kinect camera from tracking both the upper and lower extremities. It was very helpful if the participant knew the basics of how to use a computer, such as how to use a mouse and log on and off, and was able to communicate with the study team. The capacity to perform these was sometimes impacted by cognitive, communicative, or physical impairments following stroke, in which case a knowledgeable study partner was essential. It was also important that a research team member with technical knowledge be available to troubleshoot; this did not need to be in-person for most issues. The selected NIVRT platform was learned by almost all participants, showing that NIVRT is suitable for this population, given a small amount of home-based, in-person training. These findings echo those of another study; their similar home-based NIVRT system was easy to learn, enjoyable, and met their rehabilitation needs [58]. In future studies, cognitive screening (eg, requiring a Montreal Cognitive Assessment score of ≥26/30) is suggested as an inclusion criterion [59].

Adherence to both the NIVRT and iPad interventions was excellent, similar to a previous study using the Jintronix platform [60]. The NIVRT intervention was set for each participant by the intervention physiotherapist, who aimed for 30 minutes a day. The participants had limited ability to modify this, and some found the games frustrating or boring. Others found the NIVRT intervention enjoyable, which emphasizes individual differences based on one’s impairment, rehabilitation goals, and personal interests. On the other hand, participants in the iPad group were provided with a selection of suitable apps and directed to work on them for 30 minutes a day. Some participants frequently went beyond these recommendations, suggesting that they found the iPad apps to be particularly engaging, helpful, and easily accessible, more so than the NIVRT games. Indeed, the PACES showed that participants in the iPad group had greater enjoyment in their exercise program than those in the NIVRT group. There were fewer technical issues with the iPad group, and fewer complaints of boredom or repetition, which might have influenced the difference in this score. Hadjipanayi et al [61] have suggested that if NIVRT is used as a therapy rather than just for fun, the knowledge of this therapeutic purpose can diminish the enjoyment experienced and lead to boredom, which creates a challenge for NIVRT designers. On the other hand, if participants feel that the NIVRT serves a purpose (ie, enhancing stroke recovery), they may adhere even if boredom sets in, as seen in the current study. Emphasizing to patients the need for repetition of functional tasks to maximize stroke recovery is important.

Torriani-Pasin et al [62] assessed a non-NIVRT remote, asynchronous home-based exercise program for people with stroke and had an adherence rate (percent of sessions attended) of 41%. Their primary barrier was an inability to customize the exercise program to the participant. The ability to customize the Jintronix NIVRT program may have positively influenced our adherence rate. Simply directing individuals with stroke to keep exercising without a specific program or follow-up does not lead to ongoing participation in physical exercise [63], but engaging in NIVRT programs may lead to greater amounts of home-based rehabilitative exercise performed after stroke. Exercise duration is a very important influencer of adherence. Longer exercise sessions and exercising only once a week are associated with poorer adherence [64]. NIVRT has been shown to be more motivating than conventional rehabilitation [58], and most participants perceived that NIVRT enhanced their recovery and would be interested in continuing beyond the research study. Long-term adherence to the NIVRT program could be obtained by reducing the requirement to 3 times a week and encouraging other activities for variety (eg, walking, group-based exercise).

Our participants reported few adverse effects and no serious adverse events, which suggests that with safety considerations in place, NIVRT targeting balance and stepping can be done safely in the home. Our previous study investigating the use of NIVRT for individuals with mild cognitive impairment living in the community also showed that home-based NIVRT was safe [65]. Other studies on telerehabilitation after stroke with exercises performed in standing also found low rates of adverse events [17,62,66]. A Cochrane review reported no serious trial-related adverse effects using telerehabilitation after stroke [20]. The use of a reliable study partner is suggested to mitigate the risk of falls or injuries, as well as help with the technology [17].

There were no differences between groups for the physical and life participation outcome measures. This was expected, as this study was not powered to detect differences. Both groups improved significantly in many of the tests over time, which was expected, since all participants except one were in the subacute phase of their stroke recovery and were undergoing natural recovery as well as increasing their ability to adapt to the home environment soon after returning home. The NIVRT group demonstrated a clinically important improvement for the FTSTS test (ie, a decrease of more than 2.3 seconds) [67]. For the other outcome measures, changes recorded were not beyond the smallest real difference or minimal clinically important difference. Feasibility for this slate of outcome measures was shown, although two participants could not complete the TUG or FTSTS tests due to weakness. Participants using a gait aid could not carry the cup of water for the Timed Up and Go manual dual task. Of all the participants, 2 had difficulty understanding the questions in the SIS and the CIQ due to language barriers or cognitive issues.

While the use of NIVRT and similar technologies for telerehabilitation of standing balance and gait is less common than for upper extremity recovery, one group reported small but statistically significant improvements in functional gait (measured with the TUG test) and fear of falling for subacute stroke participants using a home-based augmented reality exercise program, compared to those using a similar written program with pictures [17]. There was no difference between groups on balance measures. While this study was adequately powered, the intensity (30 minutes per day, 5 days per week for 4 weeks) may have been too low for the best benefit. Our study, while underpowered, provided 6 weeks of intervention. Indeed, Kwakkel et al [68] have determined that 15 hours of additional training is required to produce a significant improvement in activities of daily living. A future, adequately powered study would establish if NIVRT is efficacious. Supporting evidence has shown that virtual reality training (immersive and nonimmersive) can have a large effect on balance (standardized mean difference 0.51, 95% CI 0.29-0.72) and a moderate effect on walking (standardized mean difference 0.31, SD 0.09-0.53) in people with chronic stroke [69]. While some have found no significant improvement in quality of life and community reintegration with NIVRT compared to conventional treatments [70], a recent review found that virtual reality (including NIVRT) outside of telerehabilitation shows a notable benefit for quality of life compared to standard rehabilitation [71]. It is important to consider these outcome measures for future studies.

This study, along with past work [17,58] supports the feasibility of using NIVRT for telerehabilitation of standing balance and gait after stroke. The next step is to develop a definitive randomized controlled trial to determine the efficacy of NIVRT in this context, using patient-oriented research considerations [57]. It will be important to have an active control group, so that interaction with the research team and rehabilitation intensity are equalized between groups, allowing for a specific assessment of the impact of NIVRT itself. The use of consistent outcome measures (including measures of impairment, function, and participation) across studies investigating stroke rehabilitation is also essential [20,72,73]. They should include a clear description of the intervention [74], follow TIDieR (Template for Intervention Description and Replication) and CONSORT recommendations to ensure validity and consistency [75,76], and incorporate cost-effectiveness assessments [20].

### Limitations

Several equity issues became apparent. As discussed above, females were underrepresented. All participants had Wi-Fi, and the majority had experience with using computers or other technologies, showing a selection bias towards those of higher socioeconomic status who were more experienced with computers. Variations in access and use of health care technologies have previously been shown to be influenced by social inequalities [77]. Therefore, the results from this study cannot be transferred to individuals with lower socioeconomic status or those who do not have Wi-Fi or previous experience with technology. While there has been an important increase in the number of older adults who own and use digital technologies, there is a wide range of digital skills and aptitudes [78,79]. Supporting participants through digital literacy programs (such as Connected Canadians) may help facilitate the use of NVIRT. Another limitation is that the participants were not tracked or restricted as to what other rehabilitation or exercises they were performing throughout the study. This could have influenced change in the outcome measures.

### Clinical Implications and Conclusions

The use of NIVRT to engage people who have had a stroke in continuing rehabilitative exercise after discharge from inpatient and outpatient stroke rehabilitation is feasible and safe, and shows potential for benefit. The results of this study support not only the ongoing investigation of the use of NIVRT for asynchronous telerehabilitation but also its immediate clinical use for some individuals. The Canadian Stroke Best Practice Recommendations Virtual Stroke Rehabilitation Interim Consensus Statement 2022 [38] strongly recommends that “virtual stroke rehabilitation should be available as an alternative or adjunct to in-person therapy” and that individual preferences should be considered.

## Appendix

Multimedia Appendix 1 CONSORT 2010 checklist.

Multimedia Appendix 2 Interview questions for participants and study partners.

Multimedia Appendix 3 Detailed demographic data for each participant.

## Abbreviations

BBS: Berg Balance Scale
CB&M: Community Balance and Mobility
CIQ: Community Integration Questionnaire
CONSORT: Consolidated Standards of Reporting Trials
FTSTS: Five Times Sit-To-Stand
NIVRT: nonimmersive virtual reality training
PACES: Physical Activity Enjoyment Scale
SIS: Stroke Impact Scale
TIDieR: Template for Intervention Description and Replication
