# Supplementary material for: Home-Based Nonimmersive Virtual Reality Training After Discharge From Inpatient or Outpatient Stroke Rehabilitation: Parallel Feasibility Randomized Controlled Trial
Source: JMIR Rehabil Assist Technol. 2025 Mar 28;12:e64729. doi: 10.2196/64729 (PMC11992496; doi:10.2196/64729)
Supplement: Multimedia Appendix 2 [file rehab_v12i1e64729_app2.docx]

**Home-based virtual reality training after discharge from hospital-based stroke rehabilitation: A feasibility study.**

Thank you for taking the time to answer some questions about your experience with the virtual reality system.

1. You used the system (insert #) times.
2. What has been your experience using the virtual reality system? [*Let the person respond. Then probe…]*

What aspects of the experience were positive?

What aspects of the experience were negative?

1. Were any exercises or games that you did with the virtual reality system particularly useful? [*If necessary, probe…*]

Why was this?

1. Do you think that using the virtual reality system helped your recovery? [*If necessary, probe…*]

Can you give some examples why or why not?

1. Do you think that it was a good idea to spend time doing virtual reality training rather than other activities? [*Let the person respond. Then probe…]*
2. Do you have any suggestions for how we could improve the use of virtual reality system as a part of a home exercise program after stroke?
3. Would you be interested in continuing to use it at home after this study is over?

What would help you to continue to use it at home?

What would be a challenge if you wanted to continue using it at home?
