# Supplementary material for: Home-Based Nonimmersive Virtual Reality Training After Discharge From Inpatient or Outpatient Stroke Rehabilitation: Parallel Feasibility Randomized Controlled Trial
Source: JMIR Rehabil Assist Technol. 2025 Mar 28;12:e64729. doi: 10.2196/64729 (PMC11992496; doi:10.2196/64729)
Supplement: Multimedia Appendix 3 [file rehab_v12i1e64729_app3.docx]

Multimedia Appendix 3: Detailed demographic data for each participant

| **Participant Number** | **Group** | **Age** | **Sex** | **Type of Stroke** | **Side of Stroke** | **Weeks Since Stroke** | **BMI (kg/m3)** | **Hand Dominance** |
| --- | --- | --- | --- | --- | --- | --- | --- | --- |
| 1 | NIVRT | 58 | Male | Ischemic | Right | 9 | 30.1 | Right |
| 2 | iPad | 24 | Female | Ischemic | Left | 26 |  | Right |
| 3 | NIVRT | 47 | Male | Ischemic | Right | 11 | 28.1 | Right |
| 4 | iPad | 73 | Female | Ischemic | Right | 81 | 27.1 | Right |
| 5 | iPad | 66 | Male | Ischemic | Left | 5 | 29.4 | Right |
| 6 | iPad | 38 | Male | Ischemic | Right | 10 | 22.0 | Left |
| 7 | NIVRT | 67 | Male | Ischemic | Left | 24 | 24.8 | Right |
| 8 | NIVRT | 57 | Male | Ischemic | Left | 5 | 25.9 | Right |
| 9 | iPad | 80 | Female | Ischemic | Left | 26 | 27.9 | Right |
| 10 | NIVRT | 82 | Male | Ischemic | Left | 11 | 22.1 | Left |
| 11 | NIVRT | 57 | Male | Ischemic | Right | 8 | 29.6 | Right |
| 12 | iPad | 88 | Male | Ischemic | Right | 23 | 31.0 | Right |
| 13 | NIVRT | 65 | Male | Hemorrhagic | Left | 17 | 22.4 | Right |
| 14 | NIVRT | 54 | Female | Hemorrhagic | Left | 27 | 25.2 | Left |
| 15 | iPad | 51 | Male | Ischemic | Left | 10 | 33.7 | Right |
| 16 | iPad | 66 | Male | Ischemic | Left | 14 | 26.2 | Right |
| 17 | NIVRT | 75 | Male | Ischemic | Right | 28 | 21.0 | Right |
| 18 | iPad | 63 | Male | Ischemic | Right | 21 | 22.9 | Right |
| 19 | NIVRT | 57 | Male | Ischemic | Right | 11 | 22.9 | Right |
| 20 | NIVRT | 83 | Male | Hemorrhagic | Right | 15 | 21.3 | Right |
